# A survey dataset to evaluate the changes in mobility and transportation due to COVID-19 travel restrictions in Australia, Brazil, China, Ghana, India, Iran, Italy, Norway, South Africa, United States

**DOI:** 10.1016/j.dib.2020.106459

**Published:** 2020-10-24

**Authors:** Diego Maria Barbieri, Baowen Lou, Marco Passavanti, Cang Hui, Daniela Antunes Lessa, Brij Maharaj, Arunabha Banerjee, Fusong Wang, Kevin Chang, Bhaven Naik, Lei Yu, Zhuangzhuang Liu, Gaurav Sikka, Andrew Tucker, Ali Foroutan Mirhosseini, Sahra Naseri, Yaning Qiao, Akshay Gupta, Montasir Abbas, Kevin Fang, Navid Ghasemi, Prince Peprah, Shubham Goswami, Amir Hessami, Nithin Agarwal, Louisa Lam, Solomon Adomako

**Affiliations:** a Department of Civil and Environmental Engineering, Norwegian University of Science and Technology, Høgskoleringen 7A, Trondheim, 7491, Trøndelag, Norway,; b Chang'an University, School of Highway, Nan Er Huan Road (Mid-section), Xi'an, 710064, Shaanxi, China,; c Italian Society of Cognitive Behavioral Therapy (CBT-Italy), Mannelli St. 139, Florence, 50132, Toscana, Italy; d Centre for Invasion Biology, Department of Mathematical Sciences, Stellenbosch University, Matieland, 7602, South Africa; e Biodiversity Informatics Unit, African Institute for Mathematical Sciences, Cape Town 7945, South Africa,; f Department of Civil Engineering, Federal University of Ouro Preto, Rua Nove, Bauxita, Ouro Preto, 35400-000, Minas Gerais, Brazil; g Department of Geography, University of KwaZulu-Natal, Howard College City, Durban, 4000, KwaZulu, South Africa; h Department of Civil Engineering, Indian Institute of Technology Guwahati, IIT Guwahati, Guwahati, 781039, Assam, India; i State Key Laboratory of Silicate Materials for Architectures, Wuhan University of Technology, Luoshi road 122, Wuhan, 430070, Hubei, China; j Department of Civil and Environmental Engineering, University of Idaho, 875 Perimeter Drive, Mailstop 1022, Moscow, 83844, Idaho, United States; k Department of Civil Engineering/Russ College of Engineering & Technology, Ohio University, 28 W. Green Drive, Athens, 45701, Ohio, United States; l Sun Yat-sen University, School of Civil Engineering, Xingang Xi Road 135, Guangzhou, 510275, Guangdong, China; m Chang'an University, School of Highway, Nan Er Huan Road (Mid-section), Xi'an, 710064, Shaanxi, China; n Department of Geography, Lalit Narayan Mithila University, Darbhanga, 846004, Bihar, India,; o University of Connecticut, Connecticut Transportation Safety Research Center, 270 Middle Turnpike, Unit 5202 Longley Building, Storrs, 06269, Connecticut, United States; p Department of Civil and Environmental Engineering, Norwegian University of Science and Technology, Høgskoleringen 7A, Trondheim, 7491, Trøndelag, Norway; q Bam University of Medical Sciences, School of Medicine, Bam, 76615-336, Kerman, Iran; r China University of Mining and Technology, School of Mechanics and Civil Engineering, Daxue Road 1, Xuzhou, 22116, Jiangsu, China; s Department of Civil Engineering, Transportation Engineering Group, Indian Institute of Technology Roorkee, 321-A&B, Roorkee, 247667, Uttarakhand, India; t Department of Civil and Environmental Engineering, Virginia Tech, 301-D3 Patton Hall, Blacksburg, 24061, Virginia, United States; u Department of Geography, Environment, and Planning, Sonoma State University, 1801 East Cotati Avenue, Rohnert Park, 94928, California, United States; v Department of Civil Chemical Environmental and Materials Engineering, University of Bologna, Viale del Risorgimento, 2, Bologna, 40136, Emilia-Romagna, Italy; w Department of Social Policy Research Centre, University of New South Wales, John Goodsell Building, Kensington, Sydney, 2052, New South Wales, Australia; x Department of Civil Engineering, Indian Institute of Science Bangalore, C V Raman Avenue, Bangalore, 560012, Karnataka, India; y Department of Civil and Architectural Engineering, Texas A&M University – Kingsville, 917 W. Ave B, Kingsville, 78363, Texas, United States; z Department of Civil & Coastal Engineering, University of Florida, 2100 NE Waldo Rd., Sta 106, Gainesville, 32609, Florida, United States; $ Federation University Australia, School of Health, 72-100 Clyde Rd, Berwick, 3806, Victoria, Australia; # Department of Engineering and Science, University of Agder, Jon Lilletuns vei 9, Grimstad, 4879, Agder, Norway

**Keywords:** Survey data, COVID-19, Mobility, Transportation, Travel behavior, Modal share, Risk perception

## Abstract

COVID-19 pandemic has heavily impacted the global community. To curb the viral transmission, travel restrictions have been enforced across the world. The dataset documents the mobility disruptions and the modal shifts that have occurred as a consequence of the restrictive measures implemented in ten countries: Australia, Brazil, China, Ghana, India, Iran, Italy, Norway, South Africa and the United States. An online questionnaire was distributed during the period from the 11st to the 31st of May 2020, with a total of 9 394 respondents. The first part of the survey has characterized the frequency of use of all transport modes before and during the enforcement of the restrictions, while the second part of the survey has dealt with perceived risks of contracting COVID-19 from different transport modes and perceived effectiveness of travel mitigation measures. Overall, the dataset (stored in a repository publicly available) can be conveniently used to quantify and understand the modal shifts and people's cognitive behavior towards travel due to COVID-19. The collected responses can be further analysed by considering other demographic and socioeconomic covariates.

## Specifications Table

**Table:** 

Subject	Social Sciences
Specific subject area	Mobility, Transportation, Modal share, Perceived risk
Type of data	Primary data, Table
How data were acquired	The data were obtained from a web-based survey created on two platforms: Google Forms (English, Italian, Norwegian, Persian, Portuguese languages) and WenJuanXing (Chinese language). The survey, promoted on both professional and social networks, is available in English in the data repository.
Data format	Raw Analyzed
Parameters for data collection	The survey data were collected from 9 394 respondents older than 18 years old having internet access
Description of data collection	The web-based survey was promoted using a combination of snowball and purposive techniques
Data source location	Countries: Australia, Brazil, China, Ghana, India, Iran, Italy, Norway, South Africa and the United States
Data accessibility	Dataset is uploaded on Harvard Dataverse
	Repository name:
	Mobility and perceived risk associated to mobility in Australia, Brazil, China, Ghana, India, Iran, Italy, Norway, South Africa, USA before and during COVID-19 restrictions
	Data identification number: https://doi.org/10.7910/dvn/eiquga
	Direct URL to data: https://doi.org/10.7910/dvn/eiquga

## Value of the Data

• The data are related to the mobility disruptions for all transport modes (walk, bicycle, motorcycle, car driven alone, car driven in company, bus, subway, tram, train, ferry, airplane) occurring during the COVID-19 restrictions as experienced by a large survey pool comprising 9 394 respondents located in ten countries on six continents.
• The data can be useful for transport practitioners and policy makers to develop mobility strategies and intervention mechanisms to tackle the COVID-19 crisis and future pandemics facilitating the interventions according to a user's perspectives.
• The data can be used to thoroughly quantify the transport disruptions, the modal share and the cognitive behavior towards travel related to the COVID-19 mobility restrictions. A software for statistical analysis can be efficiently employed to delve into the dataset.

## 1 Data Description

The COVID-19 pandemic is a major challenge for the entire global community [1,2], as both the amount of confirmed cases and the death toll are rising at a staggering rate at this moment [3]. The two most important causes that have led to the large spread of the new respiratory syndrome are its high transmissibility and our hypermobile society [4,5]. Due to the lack of any vaccine, unprecedented measures promoting social distancing and reducing individual mobility have been enforced worldwide in the attempt to contain the pandemic [6,7]. Notwithstanding the “stay-at-home” message promoted globally, it is unclear to what extent individuals have modified their travel attitudes and behaviours in response to the bans on free movement [8]. The greatest risk for contracting and transmitting infectious diseases, for instance from shared travel modes, originates in the fact that people are in proximity in a closed environment [9]. As mobility is intimately related to habits and routines, the mitigation measures and perceived risks can entail structural alterations among all transport modes [10], [11], [12].

**Fig. 1:**
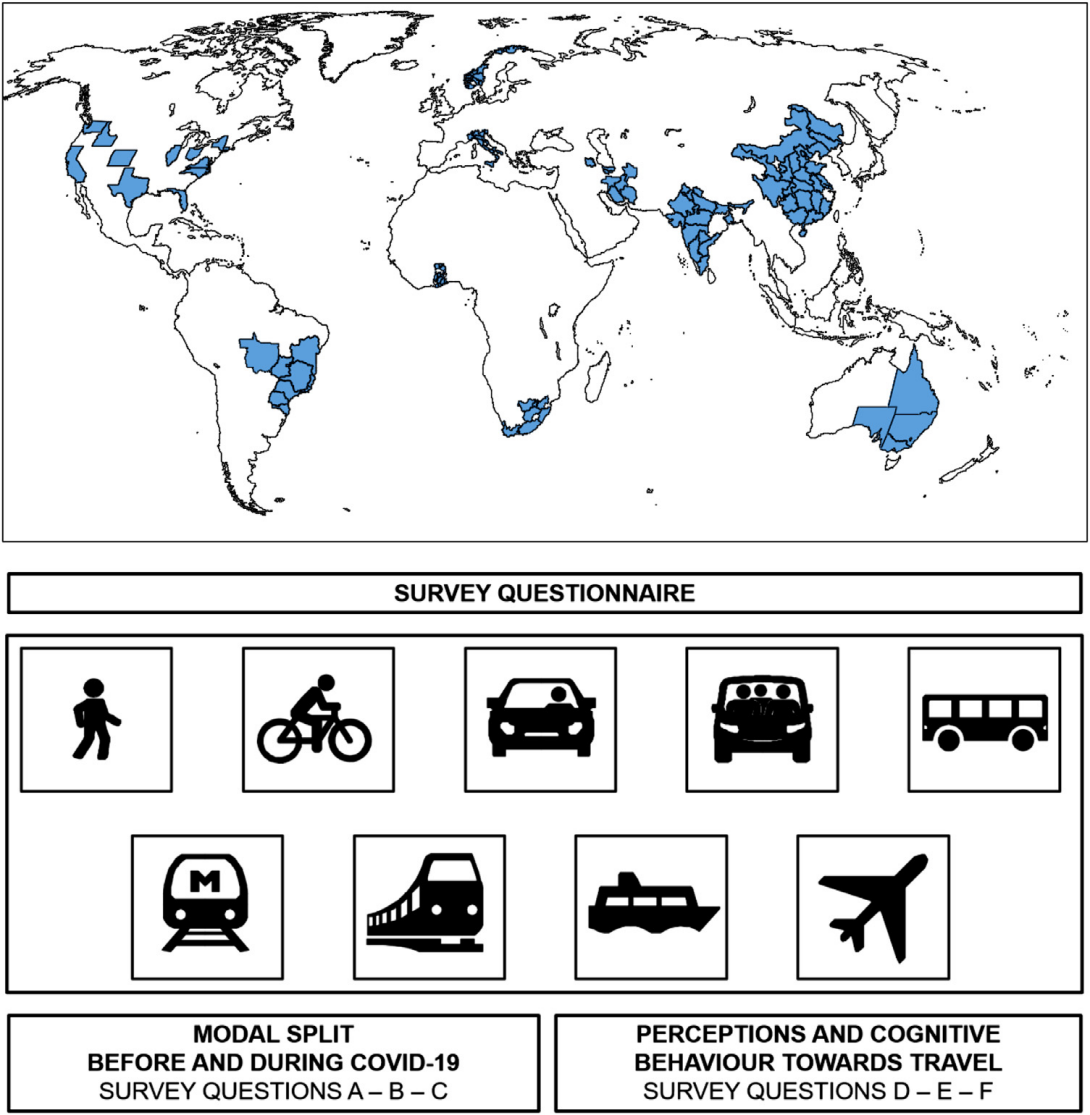
Geographical distribution of the survey respondents and schematic overview of the data collected.

**Fig. 2:**
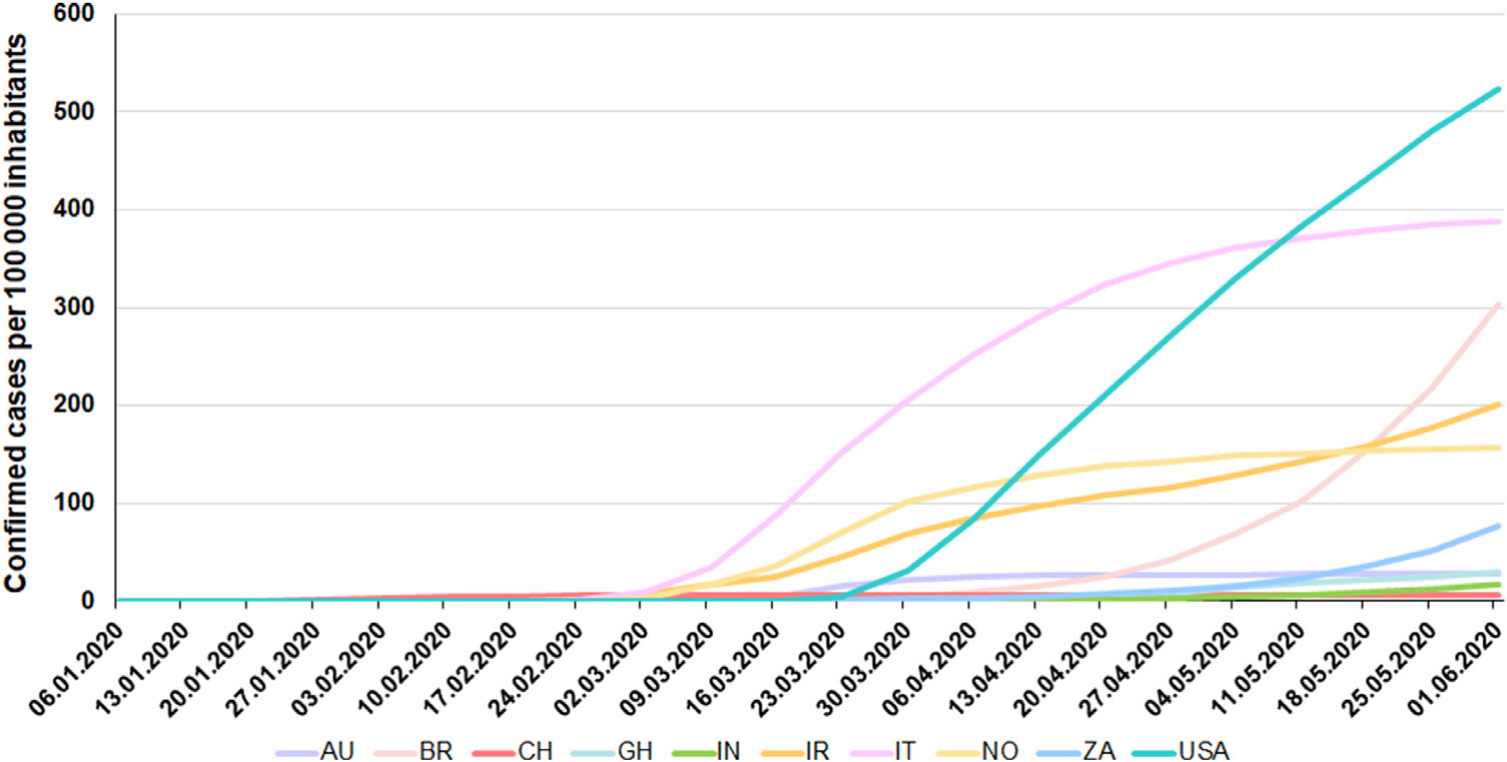
Number of COVID-19-related confirmed cases per 100 000 inhabitants in each surveyed country [3].

This survey dataset gathers information regarding the travel habits of individuals in ten countries: Australia, Brazil, China, Ghana, India, Iran, Italy, Norway, South Africa and the United States (hereafter, also referred to by their acronyms AU, BR, CH, GH, IN, IR, IT, NO, ZA and USA, respectively). An online survey was distributed during the period from the 11st to the 31st May 2020. By this time, all the investigated countries had undergone travel restrictions [13]; therefore, it was possible for all the 9 394 respondents to compare their mobility habits concerning both “before” and “during” pandemic scenarios, as well as perceived risks of contracting COVID-19 from different transport modes.

**Fig. 3:**
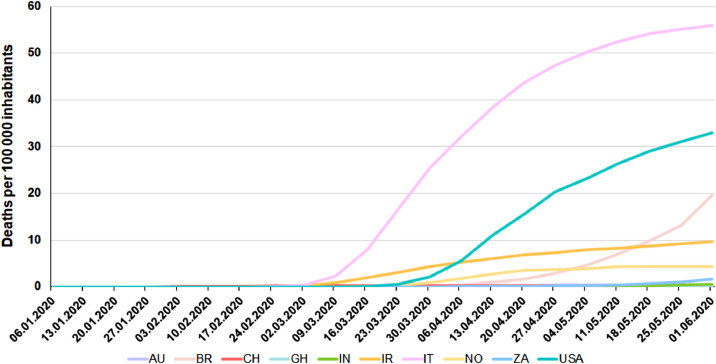
Number of COVID-19-related deaths per 100 000 inhabitants in each surveyed country [3].

**Fig. 4:**
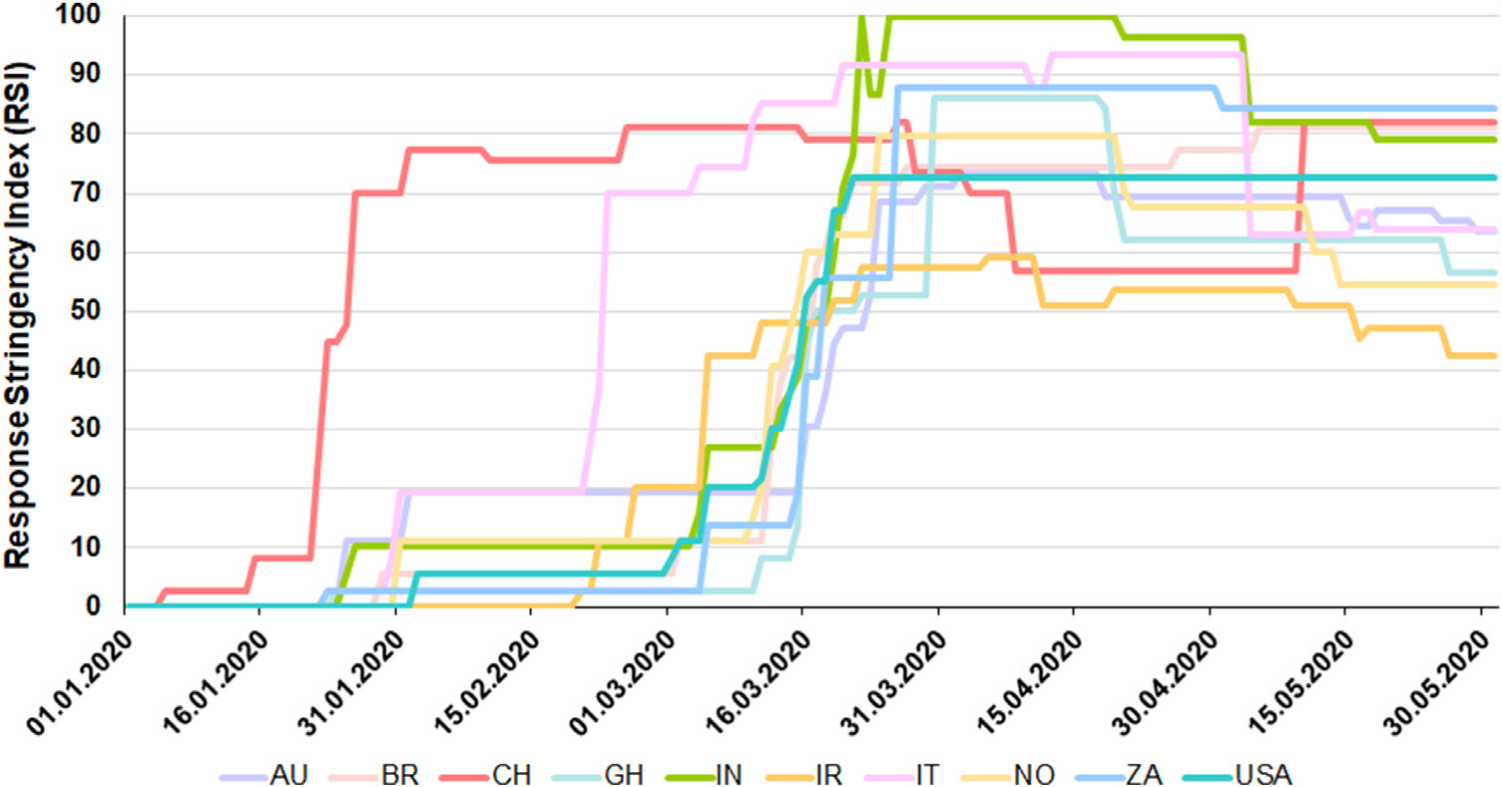
Response Stringency Index (RSI) for each surveyed country [13].

The dataset deriving from this cross-country investigation addressed two main areas: (i) to characterize the use of all transport modes (walk, cycle, car-driving alone, car-driving in company, bus, subway/tram, train, ferry, airplane) before and during the restrictions and (ii) to assess the associated perceived risk of contracting the virus and the perceived effectiveness of the travel mitigation measures. The survey targeted these two objectives with six questions [14], grouped as Question A, B, C and Question D, E, F, respectively. The cross-country harmonised dataset can be useful for many purposes comparing the use of all transport modes before and during the mobility restrictions for each activity (work/education commuting, free-time and leisure travels), assessing perceptions involving risks of contracting COVID-19 from different transportation systems and explaining cognitive and behavioural changes during the pandemic with demographic, socioeconomic and health factors. Fig. 1 displays an overview of the study and all the regions/provinces/states/counties where survey respondents have been located. In addition, temporal information regarding the number of confirmed cases and deaths are reported in Fig. 2, Fig. 3, respectively [3]. Additional information that can be useful to characterize the dynamics of the pandemic is the Response Stringency Index (RSI) [13], which is associated to the quality of the pandemic-related policies enforced in each country and is expressed by a value ranging from 0 to 100 (Fig. 4).

The dataset is publicly available on Harvard Dataverse (https://doi.org/10.7910/dvn/eiquga). The records are stored as a single file “Mobility_10_countries.xlsx” containing ten spreadsheets referring to each country; all the responses have been translated into English. Columns A and B report the respondents’ agreement to the processing of personal data and to take part in the survey. Columns C, D, E and F contain demographic information of the survey participants, namely age, gender, geographical location and highest degree, respectively. Columns G to AB and AC to AV display the frequency of use of each transport mode for work/education commuting (Question A) and free-time (Question B) travels, respectively, including information related to both before and during the enforcement of the travel restrictions. Specifically, columns S to AB are left blank if the respondent has totally or partially worked/studied remotely online due to the mobility restrictions (information reported in column R). Columns AW to BD display the frequency of leisure and shopping travels (Question C). The responses related to the perceived risks of contracting COVID-19 for each transport mode (Question D), the perceived effectiveness of the restrictive measures (Question E) and the expected time necessary for the transportation sector to recover (Question F) are reported in columns BE to BN, columns BO to BX and columns BY to CA, respectively. Responses to Question A, B, D, E are specified for each transport mode: walk, cycle, car-driving alone, car-driving in company, bus, subway/tram, train, ferry and airplane. Furthermore, the transcript of the English version of the survey “Survey_structure.pdf” is enclosed to facilitate the general overview, its content is also reported in Table 1.

**Fig. 5:**
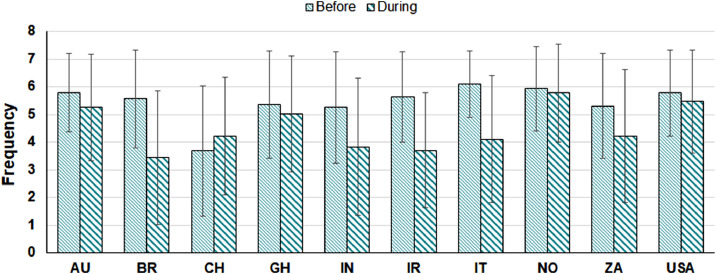
Change in mobility frequency due to the travel restrictions according to the scale “1=never”, “2=less than 1 time/month”, “3 = 1 time/month”, “4 = 2–3 times/month”, “5 = 1 time/week”, “6 = 2–3 times/week”, “7=more than 3 times/week”; mean values and standard deviations for “walk” transport mode (Question B).

**Fig. 6:**
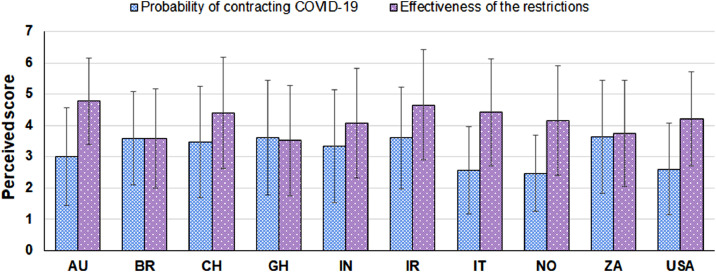
Cognitive perceptions according to the scale varying from “1=extremely low/ineffective” to “7=extremely high/effective”; mean values and standard deviations for “walk” transport mode (Question D and Question E).

**Fig. 7:**
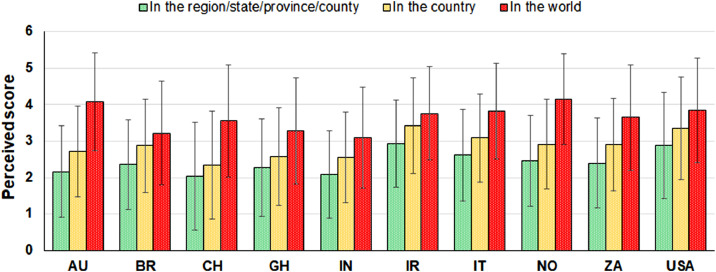
Expected time necessary for the transportation sector to recover according to the scale “1=less than 3 months”, “2=between 3 and 6 months”, “3=between 6 and 12 months”, “4=between 12 and 18 months”, “5=between 18 and 24 months”, “6=more than 24 months”; mean values and standard deviations (Question F).

Table 2 displays the demographic information (gender, age and education) of the respondents. Overall, there is a balance between male and female participants, the average age is 33 and the general education background is high, as 81.2% of the respondents hold at least a BSc degree. Even if no specific information about income was collected, and in light of the fact that the survey participants were mostly young-aged and well educated, the overall dataset is more likely to express behaviours and attitudes among upper classes. Table 3 reports on the Cronbach's alpha for the items listed in each survey question, the values indicate the reliability of the dataset. All the survey responses can be efficiently portrayed in terms of mean values and standard deviations. For example, referring to Question B, the frequency for the transport mode “walk” for each country before and during the application of the travel restrictions is reported in Fig. 5; similar graphs can be plotted for all transport modes (Question A, B, C). The data referring to the perceptions of risks (Questions D) and the effectiveness of mitigation measures (Questions E) are displayed in Fig. 6. The responses regarding the expected time needed for the transport sector to recover (Questions F) are depicted in Fig. 7.

**Table 1:** Survey structure.

**PART 1 out of 14**
**The impact of COVID-19 on mobility**
**PART 2 out of 14**
**Consent form to data confidentiality and data processing**
1. I agree to the processing of personal data and to take part in the survey
(answers: *AGREE, DO NOT AGREE*)
**Part 3 out of 14**
**Demographic information**
1. Age (i.e., 25)
(open answer)
2. Gender
(answers: *male, female, other*)
3. Region/Province/State/County where you currently stay
(answers: *choose one from the region/province/state/county list for each country*)
4. Highest degree
(answers: *primary school, middle school, high school, bachelor's degree, master's degree, Ph.D., other*)
**Part 4 out of 14 – “Question A”**
**Work/Study mobility before the epidemic**
1. Distance (in kilometers) from your living place to the place where you work/study
(open answer)
2. How often did you ONLY WALK to your workplace/school?
(answers: *7 days per week, 6 days per week, 5 days per week, 4 days per week, 3 days per week, 2 days per week, 1 day per week, 2 or 3 times per month, 1 time per month, less than 1 time per month, never*)
3. Considering ALL the transportation modes listed below to reach your workplace/school, how often did you use … ?
(answers: *7 days per week, 6 days per week, 5 days per week, 4 days per week, 3 days per week, 2 days per week, 1 day per week, 2 or 3 times per month, 1 time per month, less than 1 time per month, never, I do not have one*)
- bicycle
- motorbike/moped/quad
- car (driving alone)
4. Considering ALL the transportation modes listed below to reach your workplace/school, how often did you use … ?
(answers: *7 days per week, 6 days per week, 5 days per week, 4 days per week, 3 days per week, 2 days per week, 1 day per week, 2 or 3 times per month, 1 time per month, less than 1 time per month, never*)
- car (with someone else)
- bus
- subway/tram
- train
- ferry
- airplane
**Part 5 out of 14**
**Working remotely (online)**
1. Do you study or work from home (smart working) as a consequence of the COVID-19 outbreak?
(answers: *yes [skip part 6], no, partly*)
**PART 6 out of 14 – “Question A”**
**Work/Study mobility during the epidemic**
1. How often do you ONLY WALK to your workplace/school?
(answers: *7 days per week, 6 days per week, 5 days per week, 4 days per week, 3 days per week, 2 days per week, 1 day per week, 2 or 3 times per month, 1 time per month, less than 1 time per month, never*)
2. Considering ALL the transportation modes listed below to reach your workplace/school, how often do you use … ?
(answers: *7 days per week, 6 days per week, 5 days per week, 4 days per week, 3 days per week, 2 days per week, 1 day per week, 2 or 3 times per month, 1 time per month, less than 1 time per month, never, I do not have one*)
- bicycle
- motorbike/moped/quad
- car (driving alone)
3. Considering ALL the transportation modes listed below to reach your workplace/school, how often do you use … ?
(answers: *7 days per week, 6 days per week, 5 days per week, 4 days per week, 3 days per week, 2 days per week, 1 day per week, 2 or 3 times per month, 1 time per month, less than 1 time per month, never*)
- car (with someone else)
- bus
- subway/tram
- train
- ferry
- airplane
**Part 7 out of 14 – “Question B”**
**Free time mobility before the epidemic**
1. How often did you go out for a walk or to do sports?
(answers*: more than 3 times per week, 2 or 3 times per week, 1 time per week, 2 or 3 times per month, 1 time per month, less than 1 time per month, never*)
2. How often did you use a … ?
(answers: *more than 3 times per week, 2 or 3 times per week, 1 time per week, 2 or 3 times per month, 1 time per month, less than 1 time per month, never, I do not have one*)
- bicycle
- motorbike/moped/quad
- car (driving alone)
3. How often did you use a … ?
(answers: *more than 3 times per week, 2 or 3 times per week, 1 time per week, 2 or 3 times per month, 1 time per month, less than 1 time per month, never*)
- car (with someone else)
- bus
- subway/tram
- train
- ferry
- airplane
**Part 8 out of 14 – “Question B”**
**Free time mobility during the epidemic**
1. How often do you go out for a walk or to do sports?
(answers*: more than 3 times per week, 2 or 3 times per week, 1 time per week, 2 or 3 times per month, 1 time per month, less than 1 time per month, never*)
2. How often do you use a … ?
(answers: *more than 3 times per week, 2 or 3 times per week, 1 time per week, 2 or 3 times per month, 1 time per month, less than 1 time per month, never, I do not have one*)
- bicycle
- motorbike/moped/quad
- car (driving alone)
3. How often do you use a … ?
(answers: *more than 3 times per week, 2 or 3 times per week, 1 time per week, 2 or 3 times per month, 1 time per month, less than 1 time per month, never*)
- car (with someone else)
- bus
- subway/tram
- train
- ferry
- airplane
**Part 9 out of 14 – “Question C”**
**General mobility before the epidemic**
1. … how often did you go out and … ?
(answers*: more than 3 times per week, 2 or 3 times per week, 1 time per week, 2 or 3 times per month, 1 time per month, less than 1 time per month, never*)
- visit family members/relatives
- hang out with friends
- buy essential goods
- buy nonessential goods
**Part 10 out of 14 – “Question C”**
**General mobility during the epidemic**
1. … how often do you go out and … ?
(answers*: more than 3 times per week, 2 or 3 times per week, 1 time per week, 2 or 3 times per month, 1 time per month, less than 1 time per month, never*)
- visit family members/relatives
- hang out with friends
- buy essential goods
- buy nonessential goods
**Part 11 out of 14 – “Question D”**
**Perceptions related to COVID-19 and transportation system**
1. … how would you rate the PROBABILITY OF CONTRACTING COVID-19 from the use of the transportation modes listed below?
(answers: *extremely low, very low, low, average, high, very high, extremely high*)
- walk
- bicycle
- motorbike/moped/quad
- car (driving alone)
- car (with someone else)
- bus
2. … how would you rate the PROBABILITY OF CONTRACTING COVID-19 from the use of the transportation modes listed below?
(answers: *extremely low, very low, low, average, high, very high, extremely high, not available in the region/province/state/county where I am*)
- subway/tram
- train
- ferry
- airplane
**Part 12 out of 14 – “Question E”**
**Perceptions related to COVID-19 and transportation system**
1. … how would you rate YOUR REGION/PROVINCE/STATE/COUNTY'S RESTRICTIONS on the transportation modes listed below to limit the COVID-19 spread?
(answers: *extremely ineffective, very ineffective, ineffective, average, effective high, very effective, extremely effective, there are no restrictions in the region/province/state/county where I am*)
- walk
- bicycle
- motorbike/moped/quad
- car (driving alone)
- car (with someone else)
- bus
2. … how would you rate YOUR REGION/PROVINCE/STATE/COUNTY'S RESTRICTIONS on the transportation modes listed below to limit the COVID-19 spread?
(answers: *extremely low, very low, low, average, high, very high, extremely high, there are no restrictions in region/province/state/county where I am, not available in the region/province/state/county where I am*)
- subway/tram
- train
- ferry
- airplane
**Part 13 out of 14 – “Question F”**
**Perceptions related to COVID-19 and transportation system**
1. How long do you think it will take FOR THE TRANSPORTATION SYSTEM “to go back to normal” … ?
(answers: *between 1 and 3 months, between 3 and 6 months, between 6 and 12 months, between 12 and 18 months, between 18 and 24 months, more than 24 months*)
- in the region/province/state/county where you currently stay
- in your country
- in the world
2. Do you have any thoughts or comments that you would like to share at the end of this survey?
(open answer)
**P**art **14 out of 14**
**The survey has ended**

**Table 2:** Demographics and educations of the respondents.

	N	Gender (%)	Age		Highest education (%)				
		male/female/ other	Mean±*s*.d.	middle school	high school	BSc	MSc	PhD	other
**AU**	387	26.4/73.6/0.0	33.0 ± 11.0	0.3	20.2	42.9	24.5	10.3	1.8
**BR**	932	39.6/60.3/0.1	30.6 ± 10.0	0.2	16.1	55.7	15.8	8.9	3.3
**CH**	1 731	60.4/39.5/0.0	28.2 ± 8.2	5.1	8.4	53.8	24.7	7.0	1.0
**GH**	437	70.3/29.7/0.0	27.8 ± 5.2	0.2	10.8	67.5	16.9	1.1	3.4
**IN**	1 334	64.9/34.9/0.2	29.8 ± 8.6	0.1	4.3	40.9	41.2	10.7	2.7
**IR**	778	42.5/57.5/0.0	28.2 ± 9.8	1.0	40.7	28.8	22.1	6.8	0.5
**IT**	604	29.5/70.4/0.2	29.5 ± 9.2	1.3	33.6	28.1	28.8	6.8	1.3
**NO**	681	47.6/52.3/0.1	37.1 ± 12.6	0.4	12.0	18.1	40.1	23.2	6.2
**ZA**	582	41.8/58.1/0.2	35.4 ± 14.1	0.0	27.8	31.8	23.4	13.9	3.1
**USA**	1 928	52.7/46.8/0.5	40.6 ± 13.1	0.1	9.8	42.1	31.8	14.2	2.1
***Total***	*9 394*	*50.9/48.9/0.2*	*32.6* *±* *11.6*	*1.2*	*15.2*	*42.3*	*28.3*	*10.6*	*2.3*

**Table 3:** Values of Cronbach's alpha (internal consistency) for the responses related to each survey question.

	Question A		Question B		Question C		Question	Question	Question
	Before	During	Before	During	Before	During	D	E	F
**Cronbach's alpha**	.839	.799	.606	.686	.747	.739	.834	.888	.820

## 2 Experimental Design, Materials and Methods

The data were collected in Australia, Brazil, China, Ghana, India, Iran, Italy, Norway, South Africa and the United States with an online survey hosted on Google Forms and WenJuanXing platforms. The same questionnaire was conveniently translated into Chinese, English, Italian, Norwegian, Persian, Portuguese and was distributed via email, social media and professional networks using a combination of purposive and snowball techniques [15]. Members of the research team shared or posted a link of the survey, along with the purpose of the study, using methods that included, but were not limited to, group lists, social media platforms and personal correspondences. Given the time-sensitive nature of this study, the non-stratified nature of the responses allowed for a relatively easy means of implementation. The dataset was generated during the period from the 11st and the 31st May 2020 (single cross-sectional survey), with daily supervision and appropriate cleaning measures implemented to remove cases of obviously unrealistic responses. The final number of accepted survey participants was 9 394. Because of the international nature of the questionnaire, the survey was designed to be flexible enough for rapid deployment and, at the same time, for application in local contexts.

Overall, the survey content was related to two topics. The first part dealt with the mobility habits before and during the enforcement of the travel restriction measures, where respondents have reported on their use of all the transport modes (Questions A, B, C). Matrix-level questions have been deployed to investigate modal share derived from three main activity patterns: work/education, free-time and leisure travels [16]. The second part collected information regarding the perceived risks regarding the probability of contracting the virus from particular transportation systems and the perceived effectiveness of travel restrictions, as well as the amount of time necessary for the transportation sector to recover (Questions D, E, F). Respondents have expressed their perceptions according to Likert scales. Overall, the survey responses referring to the condition “before” the pandemic are not likely to be biased as, notwithstanding the possible distortions or telescoped facts typical of human memory [17], [18], [19], [20], retrospective questions are deemed to be reliable up to about a year [21,22].

When employing the data for socioeconomic modeling, it is important to highlight some caveats typical of this survey investigation approach: the overall survey sample, albeit substantial, was skewed from the overall population demographic composition and, therefore, the entire survey sample should only be considered as indicative of the actual perception of the general public. Anyway, the data belonging to the overall dataset can be easily stratified to create new subdatasets if one would like to build a pool of responses meeting some given demographic criteria. Furthermore, inadequate internet connections may have been encountered in developing countries during the survey.

Finally, the survey dataset presented here is the major companion of another survey dataset referring to the perceived air quality “before” and “during” the pandemic-related restrictions [23]. As the two datasets were formed at the same time (between the 11st and the 31st May 2020) but made available at two different times due to the time needed by the research team to organize and timely present the collected responses, further analyses can be performed by considering these two datasets simultaneously.

## Ethics Statement

All the survey respondents have informed their consent before joining the questionnaire consistent with the Declaration of Helsinki.

## Declaration of Competing Interest

This research has not received any specific grant from funding agencies in the public, commercial, or not-for-profit sectors. The authors declare that they have no known competing financial interests or personal relationships which have or could be perceived to have influenced the work reported in this article.
